# Advancing Cardiorespiratory Physiotherapy Practice in a Developing Country: Surveying and Benchmarking

**DOI:** 10.1155/2019/7682952

**Published:** 2019-12-15

**Authors:** Rasha Okasheh, Emad Al-Yahya, Lara Al-Khlaifat, Nihad Almasri, Jennifer Muhaidat, Dania Qutishat

**Affiliations:** The University of Jordan, School of Rehabilitation Sciences, Physiotherapy Department, Amman, Jordan

## Abstract

Management of noncommunicable diseases requires the adoption of multidisciplinary interventions that targets the modification of risk factors. Cardiovascular and respiratory diseases are amongst the four main killers of noncommunicable diseases. Physiotherapists specializing in cardiorespiratory physiotherapy are in a critical position in the management of health behaviors associated with noncommunicable diseases. However, the current context of health service in Jordan does not provide sufficient support and recognition for the delivery of specialized physiotherapy services. *Objectives. *The primary aim of this study was to describe cardiorespiratory physiotherapy service in Jordan. The secondary aims of this study were to: (i) Identify benchmarks from international contexts and guidelines for the delivery of cardiorespiratory physiotherapy service. (ii) Identify gaps and areas for development in the current delivery of cardiorespiratory physiotherapy service. *Methods. *This two phase study included a survey and a conceptual review with benchmarking. Following ethics approval, a cross sectional survey of physiotherapists practicing in Jordan was conducted. In phase 1, a survey was developed to describe the relevant dimensions of cardiorespiratory physiotherapy service. In phase 2 a conceptual review of the literature was performed to identify domains of service delivery and criteria required for optimal delivery of cardiorespiratory physiotherapy service. In the discussion we integrated the results of the survey within the benchmarks that emerged from the conceptual review of literature in order to identify gaps and areas for development in the current delivery of cardiorespiratory physiotherapy service. *Results.* Phase 1: Data emerging from the survey suggests that Physiotherapists in Jordan lack training and autonomy, preventing them from acquiring advanced roles particularly cardiorespiratory physiotherapy that requires specialised knowledge and skills. The current delivery of the service is limited to acute interventions, and is based on limited, unstructured referral from physicians depriving the patients from the service. The context of health service in Jordan does not provide sufficient recognition for physiotherapy; neither does it support the delivery of multidisciplinary interventions by appropriate regulations and policies. Phase 2: The following three domains emerged from the literature and were used for describing cardiorespiratory physiotherapy in Jordan: people, scope of practice, and context of practice. *Conclusion. *Advancing practice requires developing competencies relevant to cardiorespiratory physiotherapy particularly diagnosis, health promotion, and prevention. It is recommended that health authorities should develop regulations and policies that promote the recognition and integration of physiotherapists in the healthcare system, as well as facilitating the implementation of patient centred, multidisciplinary interventions.

## 1. Background

Noncommunicable diseases are a global burden in the 21st century, yet mostly preventable [1]. Cardiovascular and chronic respiratory diseases are amongst the four main killers of noncummunicable disease [2]. Prevention and control of noncummunicable diseases require the adoption of resolutions related to the modification of health behaviors that are known to contribute to the etiology of these diseases such as WHO Framework Convention on Tobacco Control [3], the Global strategy on diet, physical activity, and health [4], and the Global strategy to reduce the harmful use of alcohol [5]. However, lack of interest in cardiorespiratory physiotherapy has been frequently reported in the literature [6]. Calls for promoting this speciality has been presented by Dean et al. [7] in their paper “Cardiovascular/cardiopulmonary physical therapy sinks or swims in the 21st century: Addressing the health care issues of our time”.

A special branch of physiotherapy is cardiorespiratory physiotherapy. Cardiorespiratory physiotherapy is concerned with the assessment and management of physical and functional impairment, activity limitations and participation restrictions resulting from impairment of body functions, and structures of the cardiovascular and the pulmonary system as a result of a disease, injury, or other conditions. The role of physiotherapy interventions in the management of acute and chronic cardiac and respiratory diseases has been reported in a number of evidence based guidelines and statements [8, 9]. A core component of cardiorespiratory physiotherapy interventions is exercise prescription and behaviour change. This resonates with the identified strategies for the management of noncummunicable disease [3–5].

Despite the importance of cardiorespiratory physiotherapy in the management of non communicable diseases, there remains gaps in the delivery of services in Jordan. Barriers to the provision of rehabilitation services including physiotherapy have been articulated in the “WHO global disability plan” including insufficient identification of priorities; absence of relevance of policies and plans; high costs, and lack of appropriate funding mechanisms; lack of structured training affecting the competency of professionals, shortage in logistic resources; and ineffective service models that do not support integrated, multidisciplinary practice [10]. An effective service model for the management of noncummunicable diseases requires patient-centred, integrated care plans, and multidisciplinary interventions [11]. However, it is unknown whether such model is implemented or supported in the health care system in Jordan. It is also not clear whether the healthcare system is supportive of specialist roles in physiotherapy such as cardiorespiratory physiotherapy.

A structured search returned no data on the demographic profile of physiotherapists in Jordan. No published data were retrieved after researching the website of the Jordanian physiotherapy association JPTS [12], and the ministry of health annual report [13]. Limited information was available through the world confederation of physical therapy country profile (Jordan) [14]. Data on health services provided by the ministry of health did not provide information on physiotherapy services; rather it was included under “allied health” with no further verification [13]. This emphasizes the under recognition of physiotherapy by health authorities and is detrimental to the progression of the profession and the development of specialist roles such as cardiorespiratory physiotherapy. Therefore, the primary aim of this study was to describe cardiorespiratory physiotherapy service in Jordan.

The effectiveness of cardiorespiratory physiotherapy in reducing mortality and improving quality of life following cardiac and respiratory diseases is widely recognized [8, 9]. A number of models for implantation and delivery of cardiorespiratory physiotherapy service exists internationally [9, 15–21]. It is imperative that we respond to the pressing need of developing cardiorespiratory physiotherapy in Jordan and Eastern Mediterranean regions. In order to develop this service it is important to identify gaps and devise remedial plans. Therefore, the secondary aim of this study was to:

- Identify benchmarks from international contexts and guidelines for the delivery of cardiorespiratory physiotherapy service.
- Identify gaps and areas for development in the current delivery of cardiorespiratory physiotherapy service.

In order to achieve the above aims, the study was conducted in two phases:

Phase I: a cross sectional observational study using survey method to describe cardiorespiratory physiotherapy service in Jordan.

Phase II: a conceptual review of literature [15] for the identification of a framework for organizing benchmarks from international context and guidelines.

In the discussion we have integrated the results that emerged from phase 1 within the framework that emerged from phase 2 in order identify gaps and potentials for the development in delivering cardiorespiratory physiotherapy.

## 2. Phase I: Cross Sectional Observational Study

This phase was designed and reported in accordance with STROBE (Strengthening the Reporting of Observational studies in Epidemiology) guidelines.

### 2.1. Ethics

The study was approved by The Jordan University hospital ethics committee (245/2016), and by the institutional review board at King's Hussein Cancer Centre (17KHCC94).

### 2.2. Methods

#### 2.2.1. Survey Tool Development

In order to ensure content validity; it was required to identify the information that is potentially relevant for describing the service “cardiorespiratory physiotherapy practice in Jordan” that should be explored. To achieve this, a review of international guidelines and literature that describes physiotherapy and/or cardiorespiratory physiotherapy was conducted. The search terms used are presented in [Supplementary-material supplementary-material-1].

The literature search resulted in a number of documents, webpages, and research articles that were reviewed and analyzed. The key references included:

- A core syllabus for postgraduate training in respiratory physiotherapy: European respiratory Society publication [16].
- Allied respiratory professionals: EUROPEAN LUNG white book [17].
- Respiratory Physiotherapy HERMES project analysis report of the needs assessment preliminary survey: European respiratory society publication [18].
- Concise BTS/ACPRC guidelines physiotherapy management of the adult, medical, spontaneously breathing patient: British Thoracic Society Publication [9].
- Today's Physical Therapist: A Comprehensive Review of a 21st-Century Health Care Profession: the American physical therapy association [19].
- WCPT International Confederation of Cardiorespiratory Physical Therapy general meeting report: http://www.iccrpt.org/ [20].
- Policy statement “description of physical therapy”: World confederation of physical therapy [21].

During the review, clinical responsibilities, knowledge areas, and skills that were potentially relevant to describing cardiorespiratory physiotherapy practice were identified.

A consensus was made by the research team to follow the organisation of the survey used by the European respiratory society in the Respiratory Physiotherapy HERMES project [18] with the adaptation to the context of Jordan. A web based survey was developed using “Surveymonkey” tool. The survey had six main sections.

- Background information (Respondents' characteristics).
- Clinical profile.
- Knowledge and awareness of cardiorespiratory physiotherapy.
- Current cardiorespiratory postgraduate training practices.
- Implementation and context of clinical practice.
- Clinical responsibilities.

The survey included a combination of open ended and multiple choice questions, with the possibility of choosing more than one response. The aim was to allow for adequate description of practice and to accommodate the variations in practice that could have occurred in Jordan, but not represented in the international guidelines and literature.

To further ensure validity, the survey was reviewed by:

- The research team: six assistant professors with a PhD degree in physiotherapy.
- Three stakeholders external to the research team: two of them are cardiorespiratory physiotherapy module leads in two Jordanian universities; one with a PhD degree in physiotherapy and the other with a PhD degree in exercise science and one clinician “physiotherapist”.

The review included the following activities:

- Content revision, to ensure statements reflect on current international practice of cardiorespiratory physiotherapy.
- Editorial changes to improve readability.
- Organizational changes to improve grouping of questions.

The validity of the survey was tested using a pilot. A pilot was distributed to a small group of respondents who were asked to try out the survey and evaluate the clarity of the language. Based on the pilot response the following was performed.

- A correction of a technical error encountered in survey development using the Survey Monkey.
- Adding a clarification or definition beside some statements.
- Establishing an interview based survey “same questions” alongside the web based survey.
- An Arabic version of the survey was developed (Translation was checked by two researchers. No responses were returned on this version).

#### 2.2.2. Sampling and Survey Distribution

Convenience sampling method was used. The study population was defined as physiotherapists practicing in Jordan. The number of physiotherapists registered at the Jordanian physiotherapy association was estimated to be about 1000 in the month of June 2017 (It is worth noting, that based on the number of physiotherapy graduates from the three universities in Jordan the number of legally practicing physiotherapists would be higher. However, as there is no official national record of the numbers we decided to use the estimates provided by the society, which is based on the number of registered physiotherapists.). The survey was open on survey monkey from June 2017 to June 2018. The survey was promoted on social media and though the network of the Jordanian Physiotherapy Society.

To increase representativeness, the survey was also distributed at the physiotherapy department of three major health institutions in Jordan; the university of Jordan Hospital, the King Hussein Cancer Centre, and the Al-Bashir governmental hospital.

#### 2.2.3. Analysis of the Survey Data

The analysis of the survey included a number of steps to ensure the integrity of the data. All returned surveys were checked for completeness. Reponses from ineligible participants such as countries other than Jordan, and health professionals other than physiotherapists were removed. SPSS version 22 was used to analyse the data. As the primary aim of the study was to describe cardiorespiratory physiotherapy service in Jordan, descriptive statistics were used. Frequencies and percentages were used to describe:

- Respondents' characteristics.
- Clinical profile of respondents.
- Knowledge and awareness of cardiorespiratory physiotherapy.
- Current cardiorespiratory postgraduate training practices.
- Implementation and context of clinical practice.
- Clinical responsibilities.

### 2.3. Results of the Survey

There were 84 complete responses. It was not possible to accurately report the response rate as the number of practicing physiotherapists is not officially documented. An estimate of 1000 physiotherapists was provided by the Jordanian Physiotherapy Association, as of June 2017. Based on this, the response rate is about 8%. The results are presented following the organisation of the sections in the survey.

#### 2.3.1. Respondents' Characteristics

Characteristics of respondents were described in terms of Age, Gender, geographical region, and type of practice (Table 1).

Most respondents (47.6%) were in the age group of 20–29, followed by the age group of 30–39 (36%). Of the total number of respondents 63% were females.

Considering the geographical region of practice, the majority of respondents 90.5% reported practicing in the capital of Amman. In terms of the type of practice; practicing in homes and private physiotherapy centre's was reported by 42.9% of respondents followed by “governmental” type of practice (35.7%). Only 1.2% of respondents reported working in community based rehabilitation center.

#### 2.3.2. Clinical Profile of Respondents

This section describes the clinical settings within which respondents practice, the duration of their clinical experience, the number of weekly working hours, and the area of physiotherapy specialty.

**Figure 1:**
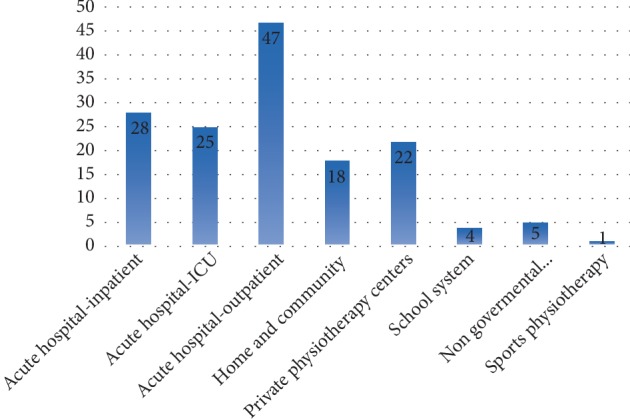
The distribution of physiotherapists amongst different clinical settings.

Figure 1 presents the distribution of physiotherapists amongst different clinical settings. Respondents were allowed to choose more than one option as it is usual for physiotherapists in Jordan to practice in more than one setting. The majority of respondents reported working in acute hospital setting; (47) in the outpatient, and (28) in the inpatient.

The clinical profile of respondents is presented in Table 2. Most respondents reported less than 11 years of experience (70.2%), only 3.6% reported more than 20 years of experience. The number of weekly working hours was reported to be 40–49 h/week by 51.2% of respondents and 30–39 h/week by 21.4% of respondents. 67.9% of respondents reported a “Bachelor degree” as the level of education. The least reported was the “Doctorate” level of education (2.4%).

**Figure 2:**
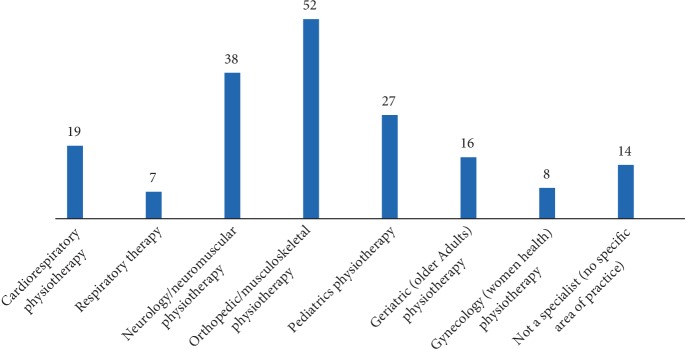
Type of speciality.

Respondents could choose more than one response for the question on specialty as there are no regulations for specializing in physiotherapy in Jordan. Most clinical settings require respondents to rotate on different specialties. “Orthopedic/musculoskeletal” (52), and “neurology/neuromuscular” (38) were the most commonly reported specialties. 19 respondents reported specializing in cardiorespiratory physiotherapy and 7 reported specializing in respiratory therapy (Figure 2).

#### 2.3.3. Knowledge and Awareness of Cardiorespiratory Physiotherapy

When asked about the difference between cardiorespiratory physiotherapy and respiratory therapy 69% of the participants reported that they had known the difference. Unfortunately, there were no further questions to verify their knowledge.

#### 2.3.4. Current Cardiorespiratory Postgraduate Training Practices

Most respondents (79.8%) reported that there is no training program for cardiorespiratory physiotherapy. Respondents reporting that there was a training program (19%) at their institution declared that this training has content related to respiratory physiotherapy only.

#### 2.3.5. Implementation and Context of Clinical Practice

This domain describes the context of cardiorespiratory physiotherapy in Jordan. Questions were asked to verify institutional practices in terms of recognition, multidisciplinary practice, and referral. Responses are presented in Table 3.

  (i) Recognition of Cardiorespiratory Physiotherapy.

Only 13.1% of participants reported that cardiorespiratory physiotherapy is recognized as a subspecialty of physiotherapy in their institution, while 10.7% reported that only respiratory physiotherapy is recognized as a subspecialty. Recognition was in the form of either postgraduate training recognized nationally, or specialisation based on job merit.

  (ii) Multidisciplinary Practice.

There were no recommendations in terms of guidelines or policies for multidisciplinary team practice in 66.7% of the reported responses.

  (iii) Referral.

Most respondents reported that there was no referral of patients with cardiorespiratory problems to the physiotherapy outpatient clinic (76.2%).

#### 2.3.6. Clinical Responsibilities

**Figure 3:**
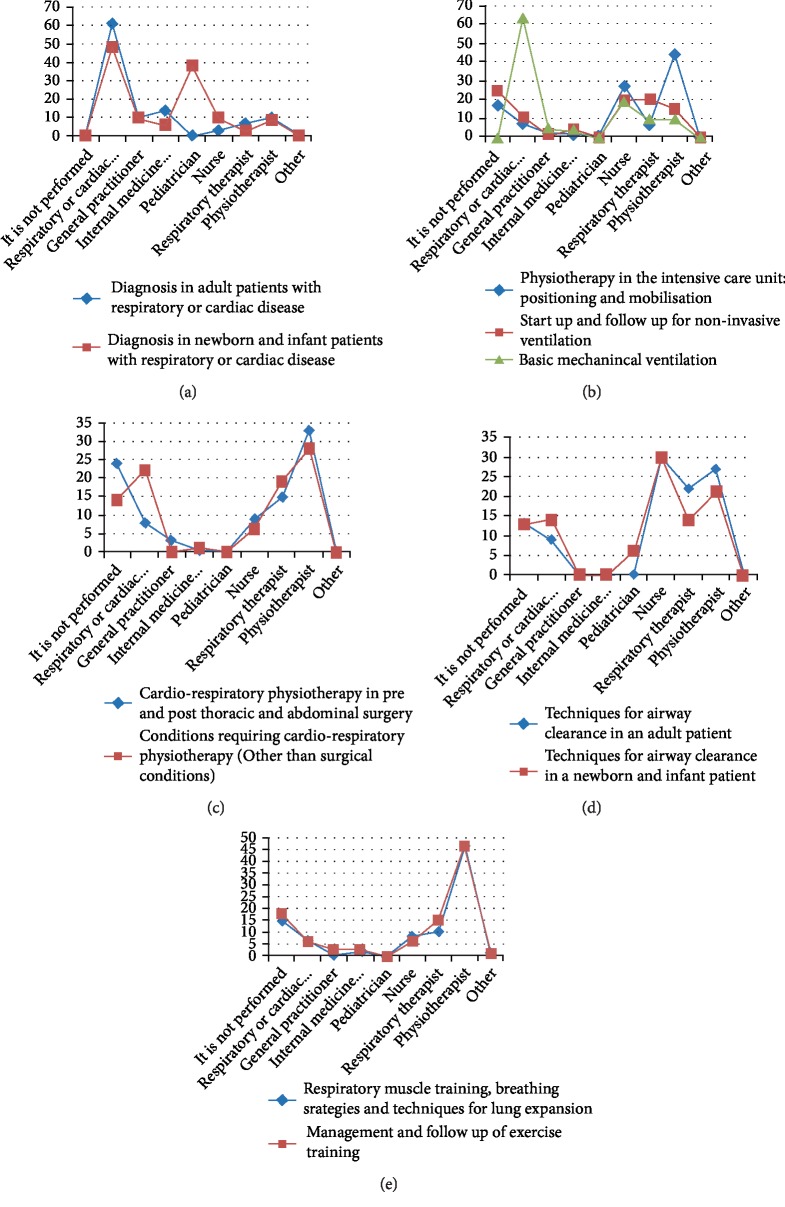
Distribution of clinical responsibilities. (a) Diagnosis. (b) Physiotherapy in the ICU. (c) Physiotherapy pre and post surgery. (d) Airway clearance. (e) Respiratory muscle training, breathing strategies and lung expansion.

This part describes the frequency of involvement of health professionals in different clinical activities related to the management of patients with cardiorespiratory problems, this is presented in Figure 3.  Figure 3(a) shows that physicians' particularly cardiac or respiratory specialists and pediatricians, in case of newborn and infants, are more likely to be involved in establishing the diagnosis.

Nurses and physiotherapists are responsible for the delivery of physiotherapy interventions in the intensive care settings (Figure 3(b)). However activities related to the delivery of basic mechanical ventilation is the responsibility of the cardiac or respiratory specialist “physician”.

Physiotherapy treatment for patients with cardiorespiratory problems, whether post surgical or not, it is mainly delivered by the physiotherapist. However, the cardiac or respiratory specialists are still involved to a lesser extent (Figure 3(c)).

Airway clearance techniques are delivered by nurses, physiotherapist, and respiratory therapists (Figure 3(d)). While there is a predominance of physiotherapists in the delivery of respiratory muscle training, breathing strategies, and techniques for lung expansion, and management and follow up of exercise training (Figure 3(e)).

## 3. Phase 2: Conceptual Review of the Literature

### 3.1. Aim

- Identify domains relevant to the description of a health service with particular reference for rehabilitation services.
- Identify criteria that represent benchmarks for the delivery and implementation of cardiorespiratory physiotherapy against which current practice in Jordan could be compared.

### 3.2. Methods

#### 3.2.1. Search Strategy and Inclusion Criteria

In order to identify domains and criteria relevant for describing the service, the following key words were used to retrieve literature:

Health service, rehabilitation, physiotherapy, evaluation, model, and delivery.

The references were included if they were reported in English.

Four researchers; RO, EA, JM, and DQ reviewed the literature. An agreement was reached to include the following references: [21–24].

#### 3.2.2. Conceptual Review of the Literature

The activity included identifying and defining concepts related to health service delivery and implementation. The process of concept identification included retrieving descriptions of quality indicators, success indicators, and domains of evaluation that could be applied to physical therapy service. This has resulted in identifying the domains of the framework.

The process of definition included the identification of the criteria that should be evident in each domain of the framework. As agreed by the four researchers the following documents were reviewed to identify the criteria for benchmarking.

- The WCPT Policy statement “Description of physical therapy” [21].
- RODAMAP East Mediterranean region WHO [23].

### 3.3. Results

The following three domains that emerged from the conceptual review (A full description of the discussion of the literature relevant to the conceptual review is beyond d the scope of this paper and could be provided upon request.) were used as a framework to organize the extracted criteria and data emerging from the survey:

- People
- Scope of practice
- Context of practice

Following is a description of the criteria required by each domain as implied in the reviewed documents:

#### 3.3.1. People

“Physical therapists are concerned with identifying and maximizing quality of life and movement potential within the spheres of promotion, prevention, treatment/intervention, habilitation, and rehabilitation. These spheres encompass physical, psychological, emotional, and social wellbeing. Physical therapists operate as independent practitioners, as well as members of health service provider teams, and are subject to the ethical principles of WCPT” [21].

Based on the above statement the following criteria were extracted:

- Autonomous: this is defined by the WCPT as: “They are able to act as first contact practitioners, and patients/clients may seek direct services without referral from another health professional” [21].
- Competent; this includes: appropriate skills and knowledge to fulfill expected roles “promotion, prevention, treatment/intervention, habilitation, and rehabilitation”.
- Able to work within a team (with other physiotherapists and other health professionals).
- Ethical practitioner (follow the ethical principles of WCPT).

#### 3.3.2. Scope of Practice

“The scope of physical therapy practice is dynamic and responsive to patient/client and societal health needs. The practice settings will vary according to whether the physical therapy is concerned with health promotion, prevention, treatment/intervention, habilitation or rehabilitation” [21].

In order to reflect the scope of practice stated by the WCPT, the scope of cardiorespiratory physiotherapy in Jordan should pose the following criteria:

- Service provided for all age groups.
- Service provided across a setting continuum (Inpatient-community).
- Service encompass a variety of clinical responsibilities reflecting roles in assessment and diagnosis, treatment/intervention, prevention, and health promotion.
- Responds to societal needs.

#### 3.3.3. Context of Practice

“Organizing people-centred integrated health services that are safe for patients and of assured quality, based on the family practice approach, at primary health care level with robust referral systems between primary health care and hospital care” [23].

Cardiorespiratory physiotherapy is delivered within the context of health system in Jordan. Therefore, it is imperative that the profession is recognised in order to facilitate implementation and delivery. Based on the above statement the following criteria should be evident in the context within which cardiorespiratory physiotherapy service is delivered.

- Recognition: a standardised procedure for the recognition of advanced roles and specialities in physiotherapy.
- Policy and regulations supports multidisciplinary practice: the existence of guidelines that facilitates multi professional practice and supports the implementation of multidisciplinary interventions such as cardiac and pulmonary rehabilitation.
- Patient centred: the organisation of service delivery and clinical responsibilities implies that it is responsive to patients and communities needs rather than practitioner led delivery.
- Referral mechanisms: robust referral mechanisms that ensure patients have access to the appropriate services.

## 4. Discussion

We have used data from the survey to describe the domains of the proposed framework as they occur in Jordan. This data is benchmarked against the criteria extracted from the literature and discussed in the context of health system in Jordan.

### 4.1. People

This domain describes physiotherapists practicing in Jordan. As the questions in the survey did not generate any data about compliance with ethical behavior, and team working, the data generated from the survey is benchmarked against the following two criteria:

- Autonomous
- Competent

The legal framework in Jordan implies limitations on the autonomy of physiotherapists. Access to physiotherapy is based on referral by physicians. Referrals are also bounded by constrains on what could be performed by physiotherapists [14].

Our data showed that cardiac and respiratory physicians are reluctant to refer critical patients (in terms of age “infants” or diagnosis “cardiac or respiratory conditions”) and they take over the responsibility of diagnosis and management. For example when asked: Who implements the techniques for airway clearance in an adult patient? There were 9 responses suggesting that the respiratory physician perform them, as compared to 14 responses suggesting that the respiratory physician perform airway clearance in infants and newborns (Figure 3(d)). Moreover, the majority of respondents reported that there was no referral of cardiorespiratory patients to the outpatient physiotherapy clinic (Table 3) implying a pattern of service delivery that is limited to the inpatient acute intervention. Consequently constraining the scope and autonomy of practicing physiotherapists.

Most participants reported less than 11 years of experience with a bachelor level of education (Table 2). This suggests the lack of senior physiotherapists (While there is no formal definition of "senior physiotherapist" the WCPT guideline for physical therapist practice specialisation describes senior physiotherapists as those with additional qualification and experience “specialisation”.) who are able to acquire advanced roles. Therefore, they are not able to manage patients with cardiac and respiratory problems who are presented with complex clinical picture and life threatening conditions. Henceforth, it is required to increase the number of competent physiotherapists capable of delivering such services. Particular knowledge in the assessment of cardiovascular and pulmonary parameters to define safe practice parameters is essential [25]. This specialised knowledge could not be attained without specialised training in cardiorespiratory physiotherapy.

Our data suggests that the majority of institutions do not provide postgraduate training in cardiorespiratory physiotherapy. In occasions where training is provided, it was limited to respiratory physiotherapy without content related to the physiotherapy management of patients with cardiac diseases. A standardised postgraduate syllabus that encompasses all the competencies required for cardiorespiratory physiotherapy should be developed and implemented. A core syllabus for post graduate training in respiratory physiotherapy developed by Respiratory Physiotherapy HERMES project could be consulted in such endeavour [16].

Cardiorespiratory physiotherapy speciality was least reported by participants. This could be explained by the lack of training and incompetency, but could also be influenced by contextual factors that are discussed later. This data along with the reported low referral of cardiorespiratory patients to the outpatient physiotherapy clinic (Table 3) implies the limited provision of cardiorespiratory physiotherapy services in Jordan.

The above discussion raises concerns about the quality of cardiorespiratory physiotherapy clinical placements experienced by undergraduate physiotherapy students. This is particularly relevant as all of the three hospitals included in the survey are recognized as training placements for undergraduate physiotherapy students from all over the country. Limitations in the experience of cardiorespiratory physiotherapy clinical placements have been reported by Lewko et al. [25]. Following an online survey of undergraduate physiotherapy training across Europe the authors reported that undergraduate respiratory physiotherapy placement is not provided for all physiotherapy students, and most importantly there is a lack of harmonized training across Europe [25]. This further emphasizes the importance of advancing cardiorespiratory physiotherapy training on the undergraduate and postgraduate level.

### 4.2. Scope of Practice

In order to reflect on the scope of practice stated by the WCPT, the scope of cardiorespiratory physiotherapy in Jordan should pose the following criteria; against which our data is benchmarked:

#### 4.2.1. Service Provided for All Age Groups

While there were no specific questions to verify the age groups of patients that physiotherapists manage, an insight could be inferred from data on speciality. Participants reported working predominantly in paediatric, musculoskeletal, and neuromuscular physiotherapy (Figure 2). They reported less involvement in cardiorespiratory and geriatric physiotherapy, suggesting a possible limited provision of physiotherapy services to older patients. An interesting finding in our data is that physicians are more likely to be involved in the management of newborn (Figures 3(a) and 3(d)) and in critical activities such as the management of basic mechanical ventilation (Figure 3(b)). This could suggest a lack of trust, by physicians in the competency of physiotherapists, emphasizing the importance of improving both training and recognition. It also provides a possible inference that cardiorespiratory physiotherapy service provision is limited to the adult patients excluding paediatrics and geriatrics populations. Future studies should further verify such inference.

#### 4.2.2. Service Provided Across a Setting Continuum (Inpatient-Community)

The type of physiotherapy intervention and activities performed differ with the setting of practice [25]. Data on the settings of practice shows that most participants reported working in acute hospital settings (more in the outpatient), followed by private physiotherapy centres (Figure 1). We previously stated that the majority of respondents reported that were no referral of patients with cardiorespiratory conditions to the outpatient physiotherapy clinic. With the majority of physiotherapist practicing in the outpatient clinic they are less likely to see those patients depriving them from the service.

Working in community based rehabilitation was least reported by participants (Table 1). This reflects the limited provision of physiotherapy services to the acute health conditions and injuries with less focus on prevention and health promotion. Contemporary definitions identify the crucial role of the physiotherapist in the management of lifestyle related condition [26]. This mandates the development of extended physiotherapy services beyond the acute care settings.

#### 4.2.3. Service Encompass a Variety of Clinical Responsibilities Reflecting Roles in Assessment and Diagnosis, Treatment/Intervention, Prevention, and Health Promotion

The data shows the limited role of physiotherapists in establishing the diagnosis. The diagnosis is mainly performed by the respiratory or cardiac physician (Figure 3(a)). This raises two concerns: First, physiotherapists are passive recipients of patients rather than collaboratively working with other health professionals.

Second, diagnose established by the physician results in the identification of the health condition “medical diagnosis”, but not functional and movement impairments. This dictates a physiotherapy management plan that is prescriptive rather than patient centred and not necessarily addressing activity limitations or participation restrictions.

As reported by participants, physiotherapists in Jordan are involved in a range of treatment activities of cardiorespiratory patients such as airway clearance, respiratory muscle training, breathing strategies, and techniques for lung expansion, management and follow up of exercise training, and positioning and mobilisation in the intensive care unit (Figures 3(b) and 3(e)). Physiotherapists were less involved in the start of and the follow-up of non invasive ventilation (Figure 3(b)). This suggests that physiotherapists actually contribute to a variety of activities related to the treatment of the cardiorespiratory patients that were identified by the European respiratory society [27].

However, this is limited to treatment rather than diagnosis, prevention, and health promotion. The literature suggests that acquiring roles related to prevention and health promotion requires extending the service beyond the acute care setting, towards secondary care and community settings [28]. It is also suggested that physical therapy service delivery should adopt a model that integrates health promotion and prevention in daily practice [28, 29]. The Council on Prevention, Health Promotion, and Wellness [30] indicated that physiotherapists should have the knowledge, skills, and competencies relevant to health promotion and prevention. Another suggested strategy to facilitate involvement in the diagnosis and health promotion was the facilitation of multidisciplinary care delivery and the implementation of multidisciplinary interventions [30]. In the context of cardiorespiratory physiotherapy practice an example of such interventions would be cardiac and pulmonary rehabilitation programmes.

#### 4.2.4. Responds to Societal Needs

The First Physical Therapy Summit on Global Health raised concerns about the proportion of physiotherapist practicing in priority areas. This is particularly relevant as cardiorespiratory physiotherapists are specialists in exercise prescription and strategies for the modification of health behaviours related to chronic cardiovascular and pulmonary diseases [26].

Our data validates this concern. Responses to questions exploring cardiorespiratory physiotherapy specialty resulted in a number of issues. When asked about specialty or preferred area of practice, cardiorespiratory was amongst the least reported along with women health and geriatric (Figure 2). This is similar to data from examination and intervention surveys of entry-level cardiovascular and pulmonary physical therapy practice in the USA [31]. Despite the fact that cardiovascular and pulmonary physical therapy was the first established physiotherapy specialty in 1978 [32], interest in the speciality is low compared to other physiotherapy specialities. As of June 2018, the American board of physical therapy specialties reported 314 cardiovascular and pulmonary specialists as compared to 14,368 orthopaedic specialists [33]. Special interest groups recognized by Jordan include neurology, orthopaedics/manual therapy, older people, paediatrics, private practitioners, and sports physical therapy [14].There are no cardiorespiratory physiotherapy special interest groups. Interest, recruitment, and retention issues in cardiorespiratory physiotherapy have been frequently reported in the literature [34–36].

The First Physical Therapy Summit on Global Health suggested that increased adoption of cardiorespiratory physiotherapy speciality could be facilitated through establishing relevant undergraduate competencies as well as postgraduate training programs [26].

### 4.3. Context of Practice

Our data is benchmarked against the following criteria of the context within which cardiorespiratory physiotherapy service is delivered.

- Recognition
- Policy and regulations supports multidisciplinary practice
- Patient centred
- Referral mechanisms

The above criteria that describe the context are discussed collectively as they are strongly interrelated. Our data describes the delivery of cardiorespiratory physiotherapy within the context of the health care system in Jordan.

It is important to emphasize that in Jordan there are no national practice guidelines to regulate or facilitate specialisation in physiotherapy. Responses to questions on recognition implied the lack of recognition of cardiorespiratory physiotherapy. Recognition was based on post graduate training recognized nationally or specialisation based on job merit (Table 3).

Current pathways of recognition are lacking in Jordan. There are variations in the training provided at different institutions. Furthermore, specialisation based on job merits is not standardised. There is no established number of years, no requirements to submit a portfolio or record of cases, and no examination. It is based on the subjective assessment of the head of the unit to decide on the distribution of roles and specialty. Our data has already presented limitations in cardiorespiratory physiotherapy clinical practice, suggesting that specialisation based on job merit does not guarantee that the physiotherapist has a significant clinical experience that could have contributed to the development of competencies required for specialisation in cardiorespiratory physiotherapy.

In order to identify appropriate procedures for recognition in Jordan a review of international cardiorespiratory physiotherapy practice has been performed and it resulted in three main pathways. Specialisation in cardiorespiratory physiotherapy (USA), harmonized postgraduate training (European Respiratory Society), and special interest or advanced practice roles (Australia and UK). The WCPT guidelines for physical therapist practice specialisation [37] acknowledged the potential benefits of specialisation in terms of improved patient care and professional status and integrity of physiotherapists. However, the guidelines implied that specialised accreditation requires the establishment of a governance board that regulates the process, develop the standards, and implement them. It was suggested that this should be best organized through the member organization [37].

In endeavouring to assign to the “specialisation” model a number of factors should be considered. Current cardiorespiratory physiotherapy practice in Jordan is heterogeneous and limited, imposing difficulties in providing training opportunities to develop competencies required for specialist roles in cardiorespiratory physiotherapy. We have earlier discussed the implications of the legal system of referral on the autonomy of physiotherapists and the limited provision of the service. Last the role of the member organization “the Jordanian physiotherapy association” in regulating and advancing practice is still limited due to the lack of assigning to membership resulting from obliviousness of the professional responsibilities and potential benefits implied. This is further complicated by deficiencies in the support and funding from health authorities due to under recognition of the role and status of physiotherapists in the health care system. Therefore, it appears that major developments should be implemented before adopting the specialisation model.

There is a platform to form special interest group as this exists for other specialties, however, given the current limitations in the role of the society in regulating practice, it is important that this step should be accompanied with establishing standardized training opportunities. Developing undergraduate competencies related to cardio respiratory physiotherapy along with a standardized comprehensive postgraduate program provide more pragmatic pathway.

The criteria of the context within which cardiorespiratory service is provided suggests the adoption of a proactive health system that is multidisciplinary, and patient centered rather than provider led. It is also required to establish a robust referral system that ensures access to services. In submitting so such model of health service delivery the following incongruities should be considered. It was previously discussed that the current pattern of service delivery is characterized by acute provision of the service with no direct access to physiotherapy services. The marginalization of the role of physiotherapist in the process of care delivery and restricting their autonomy deprives the patients from a crucial component of service required for the management of the prevailing complex and chronic health problems.

The second issue is presented in our data that suggests the lack of regulations and policies that support multidisciplinary practice (Table 3). It also showed the lack of specialised multidisciplinary programs such as cardiac and pulmonary rehabilitation. This further shift the health service paradigm from integrated, patient centered approach towards provider led care. Interestingly it was found that when there were policies and guidelines to support multidisciplinary practice, it was more likely that cardiac and pulmonary rehabilitation programs are delivered. This suggests that policies and guidelines should be developed to facilitate the implementation of multidisciplinary interventions relevant to cardiorespiratory physiotherapy such as cardiac and pulmonary rehabilitation. This mandates a collaborative national effort across academic institutions along with the health authorities and relevant stakeholders. The WCPT POLICY STATEMNT suggests that “National physical therapy associations have a responsibility to seek support for legislation/regulation/recognition” [21].

## 5. Conclusion

The current health priorities across the Middle East region require promoting non invasive interventions that address the modification of risk factors for non communicable disease “the hallmark of physical therapy”. In tackling these priorities; the current profile of physical therapy generally and cardiorespiratory physiotherapy particularly should be raised.

Our data highlighted the gap in the delivery of cardiorespiratory physiotherapy in three main domains: People, scope of practice, and context of practice. Physiotherapists lack essential competencies required for cardiorespiratory physiotherapy practice. They also lack competencies that enable them to acquire active roles in diagnosis, health promotion, and prevention. There is no standardised training relevant to cardiorespiratory physiotherapy. The role of the physiotherapist in the health care system is marginalised, with no direct access to the service. The scope of cardiorespiratory physiotherapy is limited to the acute provision with no referral of patients with cardiorespiratory problems to the outpatient physiotherapy clinic. A limited number of physiotherapists practice in community settings further restricting the scope of practice precluding roles in health promotion and prevention. The current context of practice does not support multidisciplinary care delivery. There is a lack of integration of physiotherapists in the delivery, no recognition of their role and distrust in their competencies. There is a lack of policies, regulations, and legislations necessary for the implementation of a multidisciplinary patient centered service with robust referral mechanisms.

It is suggested that developing cardiorespiratory physiotherapy in Jordan requires developing the competencies of physiotherapist at the undergraduate and post graduate level. Moreover policies and regulations are required to improve recognition and integration of physiotherapists as well as supporting multidisciplinary interventions.

## 6. Limitations

A large proportion of respondents were females (63%) a percentage approximate to that reported by the WCPT country profile (55%). Predominance of young age group could be explained by the fact that most of the sample was physiotherapists registered by the Jordanian Physiotherapy society. A trend towards increased registration in the JPTS amongst young physiotherapists is influenced by the fact that there is increased awareness among university graduates about the benefits of registration, particularly in terms of opportunities for training and continuous professional development. The WCPT country profile (Jordan) reported that there are 2,250 physiotherapists practicing in Jordan “not necessarily licensed” [14].

Another explanation for the predominance of young age group amongst the sample is the fact that the survey was publicized by indicating its importance in developing specialty cardiorespiratory physiotherapy training and practice. Older physiotherapists were less interested in such a cause, adopting more traditional roles specialising in neuromuscular or musculoskeletal physiotherapy; they were less likely to participate.

Most of the sample recruited was from the capital of Amman. While this could be a limitation, it could be verified by clarifying the following. First, beside the web based survey, visits to the major clinical sites were performed. This included the university of Jordan hospital, the Albasheer hospital and the King Hussein Cancer Centre. These are major clinical sites that receive patients from all over the country and provide a hub for leading clinical improvement and change across the country. Therefore, including staff of the physiotherapy department at these locations have enriched the data and provided a diverse perspective on practice. Moreover, these clinical sites include both an inpatient (ICU and acute care setting) and outpatient program, allowing for the exploration of various clinical activities and practices in cardiorespiratory physiotherapy. Researchers were not able to retrieve data on the distribution of physiotherapy services across the country. This highlights the need for organized effort to provide national data on physiotherapy services in the country.

Another limitation is related to the questions of the survey. Further surveys are required to verify all criteria required for advancing service delivery with more comprehensive description of the three domains.

## Figures and Tables

**Table 1:** Characteristics of respondents.

Participant characteristic	Description	
*Gender*	Frequency	Percentage
Female	53	63.1
Male	31	36.9
Total	84	100.0
		
*Age*	Frequency	Percentage
20–29	40	47.6
30–39	36	42.9
40–49	6	7.1
50–59	2	2.4
Total	84	100.0
		
*Region of practice*	Frequency	Percentage
Amman	76	90.5
Azraq	1	1.2
Irbid	2	2.4
Madaba	1	1.2
Zarqa	4	4.8
Total	84	100.0
		
*Type of practice*	Frequency	Percentage
Governmental	30	35.7
Royal Medical Services	4	4.8
Homes and private physiotherapy centre's	36	42.9
Educational institution	4	4.8
KHCC	8	9.5
NGOs	1	1.2
Community based rehabilitation centre	1	1.2
Total	84	100.0

**Table 2:** The clinical profile of respondents.

*Years in clinical experience*	Frequency	Percentage
less than 2 years	27	32.1
2–5	20	23.8
6–10	12	14.3
11–15	18	21.4
16–20	4	4.8
More than 20 years	3	3.6
Total	84	100.0

*Number of working hours/week*	Frequency	Percentage

10–19	9	10.7
20–29	8	9.5
30–39	18	21.4
40–49	43	51.2
More than 50 h	6	7.1
Total	84	100.0

*Level of education*	Count	Frequency

Diploma (3 years)	11	13.1
Bachelor's degree	57	67.9
Post baccalaureate certificate	8	9.5
Masters	5	6.0
Doctorate	2	2.4
Other	1	1.2

Total	84	100.0

**Table 3:** Context of cardiorespiratory physiotherapy practice.

*Recognition*	Frequency	Percentage
No	56	66.7
Yes	11	13.1
Do not know	8	9.5
Only respiratory physiotherapy	9	10.7
Total	84	100.0
		
*Type of recognition*	Frequency	Percentage
No answer	8	9.5
No recognition	56	66.7
Post graduate training recognised nationally	14	16.7
Specialisation based on job merit	6	7.1
Total	84	100.0
		
*Do recommendations on cardio-respiratory physiotherapy multidisciplinary team exit*	Frequency	Percentage
No	56	66.7
Yes	22	26.2
Do not know	6	7.1
Total	84	100.0
		
*Management of cardiorespiratory conditions by multidisciplinary team*	Frequency	Percentage
Mandatory	6	7.1
Optional	26	31.0
Not supported/not facilitated	52	61.9
Total	84	100.0
		
*Is there established pulmonary or cardiac rehabilitation program*	Frequency	Percentage
No	64	76.2
Yes	20	23.8
Total	84	100.0
		
Is there a referral of patients with cardiorespiratory problems to theoutpatient physiotherapy clinic	Frequency	Percentage
No	64	76.2
Yes	20	23.8

Total	84	100.0

## Data Availability

The survey data used to support the findings of this study are included within the article and in the supplementary materials. Results of the conceptual review are available upon request from the corresponding author.

## Supplementary Materials

Supplementary Materials Appendix 1: search terms used to identify literature relevant for the description of cardiorespiratory physiotherapy.
