# Supplementary material for: Advancing Cardiorespiratory Physiotherapy Practice in a Developing Country: Surveying and Benchmarking
Source: Rehabil Res Pract. 2019 Dec 15;2019:7682952. doi: 10.1155/2019/7682952 (PMC6942898; doi:10.1155/2019/7682952)
Supplement: Supplementary Materials — Appendix 1: search terms used to identify literature relevant for the description of cardiorespiratory physiotherapy. [file 7682952.f1.pdf]

**Appendix 1: Search terms used to identify literature relevant for the description of cardiorespiratory physiotherapy**<br><br>The following terms were used interchangeably	
Cardiorespiratory physiotherapy	Practice
Cardiorespiratory Physical therapy	Service
Respiratory physiotherapy	Survey
Respiratory physical therapy	Description
Cardiovascular and pulmonary physiotherapy	Characteristics
Cardiovascular and pulmonary physical therapy	Guidelines
Cardiopulmonary physiotherapy	Policies
Cardiopulmonary Physical therapy	Regulations
	Speciality
